# Injectable sustained local release doxorubicin depot technology– a promising adjuvant to systemic treatment?

**DOI:** 10.1007/s13346-025-01841-9

**Published:** 2025-04-03

**Authors:** Andrea René Jørgensen, Anders Elias Hansen, Jonas Rosager Henriksen, Maiken Stilling, Hans Christian Rasmussen, Johanne Gade Lilleøre, Magnus Andreas Hvistendahl, Josefine Slater, Elizabeth Serrano-Chávez, Jakob Hansen, Mats Bue

**Affiliations:** 1 Aarhus Denmark Microdialysis Research (ADMIRE), Orthopaedic Research Unit, J112, Aarhus University Hospital, Palle Juul-Jensens Boulevard 99, Aarhus N, 8200 Denmark; 2 Department of Clinical Medicine, Aarhus University, Aarhus N, Denmark; 3 Department of Health Technology, Section for Cell and Drug Technologies, Technical University of Denmark, Lyngby, Denmark; 4 Department of Orthopaedic Surgery, Aarhus University Hospital, Aarhus N, Denmark; 5 Department of Forensic Medicine, Aarhus University Hospital, Aarhus N, Denmark

**Keywords:** Drug depot technology, CarboCell, Doxorubicin, Microdialysis, Local concentration, Cancer treatment

## Abstract

Drug depot technologies that release chemotherapeutics locally in cancerous tissues present an intriguing strategy. This study aimed to assess the feasibility, delivery capacity, and therapeutic efficacy of a thin needle injectable doxorubicin-loaded carbohydrate-ester-based (CarboCell) depot technology. CarboCell was evaluated in three experimental setups: (A) In non-tumorous mice, release kinetics were evaluated 24 h and 48 h after a subcutaneous depot injection. (B) In mice with syngeneic CT 26 colorectal cancer, efficacy was evaluated based on tumour growth control and survival. This was done by two intratumoral injections of 50 µl CarboCell containing 1 mg/mL or 4 mg/mL doxorubicin at 5 days intervals. (C) In ten female pigs, local and distant release of doxorubicin from a 2 mg/mL doxorubicin CarboCell (2 or 4 mL) injected into tibial metaphysis was evaluated using microdialysis in nine tissue compartments. (A) Subcutaneous CarboCell depots demonstrated a sustained release of doxorubicin with (mean ± SEM) 36 ± 13% and 48 ± 20% of the loaded dose being released at 24 h and 48 h time points, respectively. (B) Intratumoral injection effectively controlled tumour growth and markedly extended the median survival time compared to the control group. (C) Doxorubicin peak drug concentrations in the metaphysis were > 0.3 µg/mL and could be quantified at least 10 mm from the application site. The systemic spill-over was minimal. Doxorubicin-loaded CarboCell proved easily administrable, maintaining antitumoral activity, good metaphyseal distribution and providing much higher local concentrations in metaphyseal bone providing high local concentrations in metaphyseal bone with a good distribution and limited systemic exposure.

**Graphical abstract:**

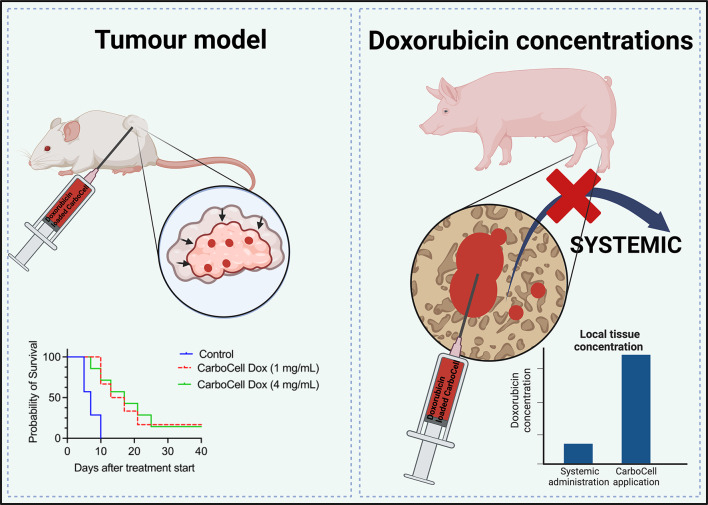

**Supplementary Information:**

The online version contains supplementary material available at 10.1007/s13346-025-01841-9.

## Highlights

Doxorubicin-loaded CarboCell has a sustained release of doxorubicin.

The cytotoxic efficacy of doxorubicin is maintained when loaded in CarboCell.

Local doxorubicin concentration after application in CarboCell is several times higher than after systemic administration.

The systemic spill-over after local application of doxorubicin-loaded CarboCell is minimal.

**Supplementary Information:**

The online version contains supplementary material available at 10.1007/s13346-025-01841-9.

## Introduction

Doxorubicin is a chemotherapeutic drug from the anthracycline family and has been used as both curative and palliative therapy for decades across several cancers including osteosarcoma [1]. Since its clinical introduction, doxorubicin and advancements in other treatment modalities have contributed to an increased 5-year survival from 30% (1960s) to approximately 80% for osteosarcoma patients today [2]. Despite these gains, there appears to be a plateau in the optimization of doxorubicin administration, illustrated by stagnating survival rates.

Traditionally, doxorubicin is administered systemically as either an intravenous bolus or continuous infusion. However, the dose and administration need to be balanced to an acceptable and tolerated level, considering effects and side-effects. The risks include general inflammation of the mucosal lining of the gastrointestinal tract, as well as the risk of leukopenia and anaemia. These factors increase the likelihood of infections and contribute to the overall malaise following systemic chemotherapeutic treatment. A particularly feared side effect of systemic doxorubicin treatment is cardiotoxicity, which depends on both the dose and the peak concentration of doxorubicin and is partly believed to be caused by its primary metabolite, doxorubicinol [2]. The maximum life-long cumulative dose of doxorubicin is often set at 550 mg/m^2^ (risk of congestive heart failure 4-26% [3–5]), but some studies have found signs of toxicity and fulminant congestive heart failure at much lower doses [3, 4]. Symptomatic cardiotoxicity can occur both acutely (during treatment) and early (within the first year after treatment) as well as up to 30 years after end of therapy. When administered as monotherapy within a dosage range of 60–75 mg/m^2^, and considering a multi-course administration, the prospects for re-administration of doxorubicin may be constrained in case of relapse. This highlights the importance of investigating newer methods of administration/application to improve potential therapeutic effects of doxorubicin treatment.

Local application of doxorubicin holds the potential to lower the systemic side effects and increase the therapeutic efficacy at target site. First, through a low systemic spill-over, and second through higher local/intra-tumoral concentrations, which is significant as the effect is believed to be correlated with the concentrations [6]. By reducing systemic toxicity, this approach may not only reduce side-effects but also create a more favourable microenvironment for a potential anti-cancer immune activation. While beyond the scope of this study, this could be particularly relevant for immunotherapy, where a robust and responsive immune system plays a critical role in cancer control.

To allow for a local administration and sustained release of doxorubicin, we developed a carbohydrate-ester-based formulation technology termed a CarboCell. The CarboCell is a low-viscosity liquid technology (a non-aqueous gel) that can be injected with thin needles into accessible tissues. Upon injection, the liquid will form positionally stable semisolid depots at the site of injection providing a sustained release of the incorporated drugs [7]. The release of drug from the depot is governed by drug diffusion in the CarboCell material and can be controlled by changing the composition. The release rate can be tailored to match a desired dose and period. Desalted doxorubicin is lipophilic and therefore soluble in the CarboCell matrix. However, in the desalted form, doxorubicin is not stable. To enhance stability and allow for solubilization in the CarboCell system, a hydrophobic docusate salt of doxorubicin was prepared, also referred to as hydrophobic ion pairing (HIP). This allows for effective solubilization in and release of doxorubicin docusate from the CarboCell composition. In this specific study, the CarboCell composition was designed to provide a sustained release that is expected to be completed within approximately seven days.

This study aimed to assess the applicability, delivery potential, and efficacy of a doxorubicin-loaded CarboCell by testing it in three experimental setups; (A) release kinetics in mice, (B) therapeutic efficacy in mice (tumour model), and (C) local and distant release of doxorubicin CarboCell 2 mg/mL (2 mL or 4 mL) injected into the cancellous bone of the tibial metaphysis in pigs.

We hypothesised that the CarboCell could provide a sustained release of doxorubicin with maintained efficacy, that cancellous bone concentrations would be higher than after systemic administration and that systemic spill-over would be minimal.

## Materials and methods

### Study location

The study was conducted at the Institute of Clinical Medicine, Aarhus University, Aarhus N, Denmark. Chemical analyses of doxorubicin from the release kinetics in mice were performed at the Department of Health Technology, Technical University of Denmark, Lyngby, Denmark. All chemical analyses of doxorubicin and the metabolite doxorubicinol from the porcine studies were performed at the Department of Forensic Medicine, Aarhus University Hospital, Aarhus N, Denmark.

### Doxorubicin CarboCell formulation

Doxorubicin-docusate HIP complex was formed from stock solutions of doxorubicin hydrochloride (13.8 mM) and sodium docusate in methanol (13.8 mM) using the Bligh-Dyer method: In a 50 mL Falcon tube, 5 mL of doxorubicin in MilliQ water was added 10 mL of co-ion solution in methanol. The solutions were shaken for 15 min at 320 rpm. Five mL chloroform was added, and the mixture was shaken vigorously at room temperature for 45 min at 480 rpm. Another 5 mL of MilliQ water and 5 mL of chloroform was added, and the tube was shaken vigorously for 5 s by vortexing followed by centrifugation at 3,000 rpm for 8 min. The solution was then phase separated and the upper phase was removed, whereafter the organic phase, containing the HIP complex, was transferred to a 50 mL glass vial of known weight. Then, the chloroform was evaporated under a gentle flow of nitrogen and the weight of the formed doxorubicin-docusate HIP complex was measured by weighting.

CarboCell formulations were prepared by mixing sucrose benzoate (SuBen) with glycerol trihexanoate (GTH) and EtOH (SuBen: GTH: EtOH (60:20:20)). The mixture was placed in an ultrasonication bath at 70–80 °C for 1–2 h and occasionally vortexed to generate homogenous solutions (CarboCell) that were subsequently stored at 4 °C until further use. The doxorubicin-docusate HIP complex was fully soluble in the CarboCell up to a tested concentration of 4 mg/mL.

CarboCell has previously been shown to be a precise injection technology that can provide controlled and sustained drug delivery [8].

### Release kinetics of doxorubicin from CarboCell (mice)

To demonstrate that the CarboCell technology provides a continuous release of doxorubicin, release kinetics from 50 µL CarboCell depots injected subcutaneously in 6 female BALB/c mice were investigated. Three mice were euthanized at 24 h and 48 h, respectively, and the CarboCell depots recovered. Depots were dissolved in 1 mL DMSO and left overnight at room temperature. The solutions were filtered using a 0.45 μm nylon filter and diluted 5x before analysis (see supplementary).

### Cytotoxic efficacy of the doxorubicin-loaded CarboCell (mice)

The therapeutic activity and performance of CarboCell doxorubicin were investigated in mice carrying syngeneic tumours. Murine colorectal cancer cells (CT26, ATCC) in serum-free media (RPMI) were injected subcutaneously (300,000 cells, right flank) in 7-8-weeks-old female BALB/cJrJ mice (Janvier). Tumours were allowed to establish for 14 days reaching a mean size of 278 ± 151 mm^2^. With the mice under general anaesthesia (Isoflurane 2%) 50 µl of CarboCell compositions containing 1 mg/mL (*N* = 6) or 4 mg/mL (*N* = 7) of doxorubicin was injected into the central part of the tumour using a 25G needle and a 1 mL syringe. A control group (*N* = 7) received no treatment. One mouse (group of 1 mg/mL) did not experience tumor take and had to be excluded, wherefore this group was reduced to *N* = 6 instead of *N* = 7. The mice were injected twice at 5 days intervals. Tumour size was measured by calliper 2–3 times per week and weight loss and well-being of the mice were continuously monitored. Tumour volume was calculated by the formula Volume = length x width^2^ × 0.5 (length and width). No mice displayed failure to thrive throughout the experiment.

### Microdialysis

Microdialysis is a sampling method allowing for the evaluation of local tissue concentrations of the unbound portion of a drug of interest [9–11]. It can therefore be used to evaluate the real-time release of doxorubicin from the CarboCell in vivo.

The microdialysis system consists of a precision pump containing a syringe with perfusion fluid, a catheter with a semi-permeable membrane and a collection vial. The collection of samples happens diffusion-driven across the membrane, but due to the continuous perfusion of the system, the concentration measured in the vial only represents a fraction of the absolute tissue concentration. This is corrected by the calculation of a relative recovery, which in the present study was found using calibration by drug (doxorubicin) [12].

All microdialysis equipment was purchased from M Dialysis AB (Stockholm, Sweden). The catheters were type 70 with membrane lengths of 20 and 30 mm and type 67 IV catheters with a 30 mm membrane. Both catheter types had a cut-off value of 20 kDa. The perfusion fluid was saline, the flow rate was set at 1 µl/min, and sample collection was done in 1.5 ml LoBind Eppendorf tubes (Eppendorf, Hamburg, Germany). This setup was chosen based on previous thorough methodological evaluations, which indicate that a traditional microdialysis vial is inadequate for collecting doxorubicin concentrations [13].

### Tissue concentrations following CarboCell application (pigs)

To evaluate release to both the immediate surrounding cancellous bone and distant tissues following application of CarboCell, ten female pigs (Danish Landrace, mean weight 75 kg (range: 71–80 kg), age 5 months) were included in the study. Before the study, the pigs were kept in pens in groups of a minimum of two pigs with a light cycle of 12 h. Straw was used as bedding and they had access to ad libitum water. Feeding was restricted (farm pig ration) to limit weight gain. On the day of transportation to the surgical facility all pigs were sedated with zoletil mix ((25 mg/ml tiletamin + 25 mg/ml zolazepam) + 6.25 ml xylazine (20 mg/ml) + 1.25 ml ketamine (100 mg/ml) + 2.5 ml butorphanol (10 mg/ml) 1 ml/10 kg)). Upon arrival at the surgical facility, the pigs were placed under general anaesthesia consisting of a combination of continuous intravenous infusion of propofol (initial dose: 40 ml/h) (Fresenius Kabi, Bad Homburg, Germany) and fentanyl (initial dose: 25 ml/h) (B. Braun, Melsungen, Germany). At the end of the observation period, euthanasia was performed with an intravenous overdose of pentobarbital.

All pigs were monitored continuously by intravenous blood pressure measurement and kept at a minimum of 65 mmHg middle arterial pressure (regulated with fluid, Trendelenburg position and/or continuous norepinephrine solution). Saturation and temperature were measured, and the latter was controlled with heated or cooled fluids, covers and ventilation. Additionally, arterial blood gas samples were taken every 2 h to monitor pH, which was within a range of pH 7.41–7.57.

#### Surgical procedures

**Fig. 1:**
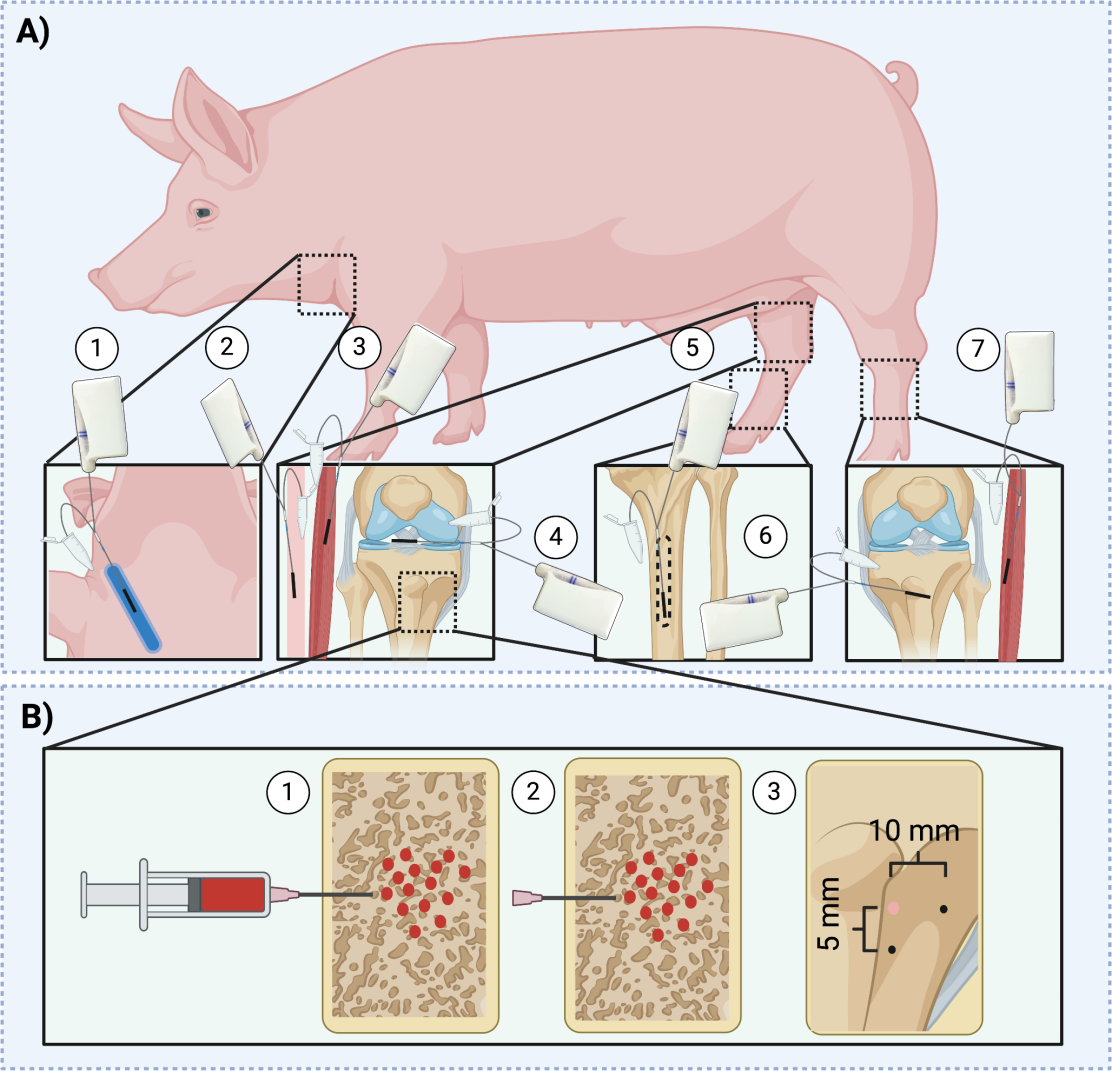
**A**) Overview of the placement of the microdialysis catheters; (1) intravenous, (2) subcutaneous tissue right hind limb, (3) muscle tissue right hind limb, (4) synovial fluid of the knee joint, (5) bone marrow, (6) cancellous bone of the left hindlimb, and (7) muscle tissue of the left hindlimb. **B**) Overview of the application of the Doxorubicin-loaded CarboCell in the cancellous bone on the right hindlimb (1,2) as well as the placement of the two cancellous microdialysis catheters (3) in the cancellous bone on the right limb, with a 5 mm and 10 mm distance to the application site, respectively

With the pig in a supine position, a central venous catheter, an arterial sheath and a type 67 catheter were placed ultrasound-guided in a jugular vein, femoral artery and intravenously, respectively (Fig. 1).

On the left hindlimb (tibia), catheters were placed as a systemic reference. A 15–20 mm incision was done perpendicular to a line approximately 10 mm distal to the epiphyseal line. A 33–35 mm (∅ 2 mm) deep drill hole was made in the cancellous bone, and a catheter with a 30 mm membrane was placed.

On both hindlimbs, 30 mm membrane catheters were placed ultrasound-guided in muscle tissue with the use of a splitable introducer (mean depth: 16 mm) and in subcutaneous tissue (mean depth: 3 mm).

On the right hindlimb (tibia), a 30 mm catheter was placed in the synovial fluid of the knee joint with the use of a splitable introducer. Additionally, an anteromedial incision approximately 10 mm distal from the epiphyseal line to the middle of the diaphysis was done. A drill hole measuring 33–35 mm (∅ 2 mm) was made in the proximal part of the diaphysis continuing longitudinally to place a 30 mm catheter intramedullary.

Prior to the placement of the two final catheters on the right hindlimb, the CarboCell was injected in the proximal tibial metaphysis. The CarboCell was loaded into two-component syringes and stored at 4 °C until 30–45 min before application and kept in the dark right up until application. A small drill hole was made in the cortical bone (∅ 1.5 mm), while the remaining passage into the cancellous bone was made with an 18G needle. The CarboCell was applied under steady pressure while removing the needle out towards the bone surface.

After application of the CarboCell, the needle was left in the injection hole and used as the guidance for a fabricated template that allowed drilling of two 23–25 mm holes (∅ 2 mm) 5 and 10 mm from the injection hole, respectively. A catheter with a 20 mm membrane was placed in each hole. After approximately 30–45 min tissue equilibration time, the sampling period was started for all catheters.

The first two pigs received administrations of 4 mL (2 mg/mL) CarboCell, but due to visible leakage from the adjacent drill holes, the remaining eight pigs received 2 mL (2 mg/mL).

#### Sampling

**Fig. 2:**
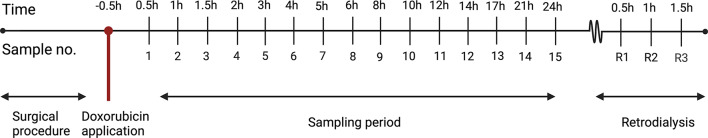
Overview of the 24 h sampling period. Time is giving above the line in hours, and samples numbers are given below the line. The numbers of the retrodialysis samples are R1-R3

Insertion of the first LoBind Eppendorf tube indicated time zero. The total observation period was 24 h. Dialysates were collected every 30 min from time 0 to 120 min, every 60 min from time 120 min to 360 min and every 120 min from time 360 min to 840 min. At time 960 min, 1200 min and 1380 min a LoBind Eppendorf was placed for collection of dialysates over 60 min (Fig. 2). At the midpoint of each dialysate sampling interval, a venous blood sample was taken. A total of 15 dialysates (from each catheter) and 15 blood samples were taken. After the 24 h observation period, a calibration period lasting 3 × 30 min (3 dialysates per catheter) was performed. During the entire observation period, the operating room was kept dark due to the risk of photodegradation of doxorubicin.

#### Handling of samples

Venous samples were collected in 4 mL EDTA 1.8 mg/mL tubes and centrifuged at 3,000 g, for 10 min at 5 °C. Plasma and dialysates were stored immediately at -80° until analysis.

#### Quantification of doxorubicin and doxorubicinol in dialysates and plasma samples from pigs by ultra-high performance liquid chromatography and tandem mass spectrometry

Doxorubicin and doxorubicinol concentrations in microdialysates and plasma samples from pigs were measured by ultra-high performance liquid chromatography and tandem mass spectrometry (UHPLC-MS/MS) as previously described in detail [13]. Briefly, the method was based on quantification using reference standard compounds and stable isotope labelled internal standard. The compounds were analyzed by UHPLC-MS/MS with reverse phase chromatography and compound detection by multiple reaction monitoring MS/MS mode. The lower limits of quantification for doxorubicin and doxorubicinol were estimated to 0.002 µg/mL (dialysate) and 0.003 µg/mL (plasma), and the standard requirements for precision (CV < 15%) and trueness (bias < 15%) were met.

### Pharmacokinetic analysis and statistics

The concentrations measured in dialysates were attributed to the midpoint of each sampling interval and represented the unbound fraction of doxorubicin. Plasma samples represented the total (protein-bound + unbound) concentrations. Due to an abundance of values below lower limit of quantification (LLOQ), the following parameters were only estimated for the two compartments, cancellous bone 5 mm and 10 mm: area under the concentration-time curve (AUC_0 − 24 h_) from time zero until 24 h and peak drug concentration (C_max_). The AUC_0 − 24 h_ was calculated using the linear up-log-down trapezoidal method. The C_max_ was calculated as the mean peak concentration of doxorubicin in each compartment. The pharmacokinetic parameters for doxorubicin between the two groups were compared in Stata (version 18.0, StataCorp, College Station, Texas, USA) using mixed models for repeated measures, followed by post-hoc tests for pairwise comparisons. All model assumptions were tested by visual inspection of residuals, fitted values, and estimates of random effects. Survival was analysed by the log-rank test. A *P*-value < 0.05 was regarded as statistically significant.

## Results

Results are presented in the same order as explained in the method section. First the effect of CarboCell is presented based on release kinetics and cytotoxic efficacy found in the mouse model. Thereafter, concentrations of doxorubicin and doxorubicinol found in the pig model are presented.

### Release kinetics of doxorubicin from CarboCell (mice)

The subcutaneous CarboCell depots displayed release at the 24 h and 48 h time points. HPLC analysis demonstrated that 36 ± 13% (mean ± SEM) had been released at 24 h and 48 ± 20% at 48 h post-injection (supplementary Figure S1).

### Cytotoxic efficacy of the CarboCell (mice)

To support that therapeutically active doxorubicin is released from CarboCell depots the therapeutic efficacy was evaluated by intratumuoral injections of 50 µl of 1 mg/mL or 4 mg/mL doxorubicin in CarboCell. The CarboCell doxorubicin displayed growth inhibition of CT26 tumours and significantly increased median survival time relative to untreated controls. There was no difference between the therapeutic activity of CarboCell doxorubicin 1 mg/mL and 4 mg/mL (supplementary Figure S2).

### Tissue concentrations following CarboCell application (pigs)

All pigs completed the study period. The range of mean relative recovery (SD) was 58% (7)– 86% (3) for the two pigs receiving 4 mL of CarboCell and 50% (13)– 82% (12) for the eight pigs receiving 2 mL. The CarboCell was easily applied in the cancellous bone, although some leakage from the drill hole was visible.

**Fig. 3:**
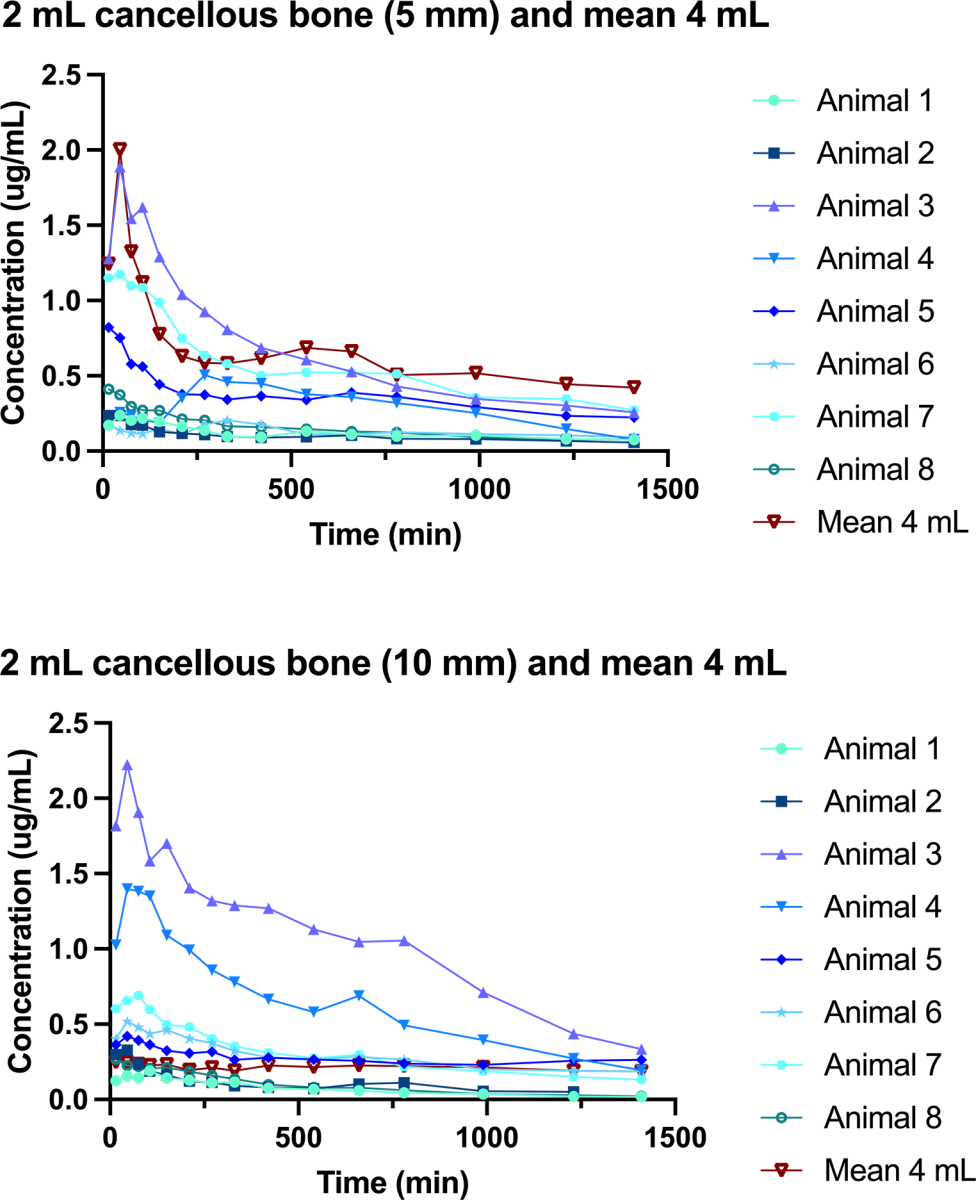
Time-concentration profiles for cancellous bone 5 mm and 10 mm. Illustrated individually for all animals receiving a 2 mL application and as a mean for the two animals receiving a 4 mL application

The time-concentration curves and pharmacokinetic parameters for cancellous bone 5 mm and 10 mm are presented in Table 2; Fig. 3. The mean AUC_0 − 24 h_ and C_max_ after application of 2 mL were similar for 5 mm and 10 mm. For all pigs, the concentrations in plasma were below the LLOQ for both doxorubicin and doxorubicinol. The remaining compartments mainly had values below the LLOQ, but 3/10 pigs did experience systemic spill-over with the highest value of 0.283 µg/mL found in muscle tissue of the left hind limb in one pig (Table 1).

**Table 1:** Descriptive overview of the non-cancellous bone compartments

Compartment	*N*	Concentrations min; max µg/mL
Bone marrow (right hindlimb)	5	1: 0.000; 0.004* 2: 0.000; 0.006* 3: 0.000; 0.006 4: 0.000; 0.042 5: 0.006; 0.037
Subcutaneous tissue	3	1: 0.000;0.004 2: 0.004; 0.103 3: 0.011; 0.072
Muscle	2	1: 0.006; 0.115 2: 0.000; 0.010
Synovial fluid of the knee joint	1	1: 0.024; 0.098
Intravenous	3	1: 0.000; 0.006 2: 0.002; 0.019 3: 0.002; 0.012
Cancellous bone left hindlimb	2	1: 0.012; 0.091 2: 0.000; 0.038
Muscle left hindlimb	2	1: 0.000; 0.006 2: 0.013; 0.283

Only data from pigs with concentrations ≥ LLOQ

*Received an application of 4 mL doxorubicin-loaded CarboCell

**Table 2:** Pharmacokinetic parameters for cancellous bone 5 mm and 10 mm

Pharmacokinetic parameters	2 mL CarboCell		4 mL CarboCell		Difference	*P*-value
AUC_0 − 24 h_, min•µg/mL (95%CI)						
Cancellous bone (5 mm)	*N* = 8	202.7 (164.0; 641.3)	*N* = 2	884.9 (407.7; 1362.2)	482.2 (-51.3; 1015.83)	0.071
Cancellous bone (10 mm)	*N* = 8	475.0 (164.2; 785.7)	*N* = 2	296.9 (-324.6; 918.5)	-178.0 (-872.9; 516.9)	0.595
C_max_, µg/mL (95% CI)						
Cancellous bone (5 mm)	*N* = 8	0.7 (0.01; 1.4)	*N* = 2	2.2 (0.8; 3.6)	1.5 (0.0001;3.0)	0.05
Cancellous bone (10 mm)	*N* = 8	0.8 (0.2; 1.3)	*N* = 2	0.3 (-0.8; 1.3)	-0.5 (-1.7; 0.7)	0.384

Area under the curve from time 0 to 24 h (AUC_0 − 24 h_); Peak drug concentration (C_max_)

AUC_0 − 24 h_ and C_max_ are given as medians

Doxorubicinol could be detected in both pigs receiving 4 mL doxorubicin-loaded CarboCell in cancellous bone 5 mm and 10 mm, range from LLOQ (0.002 ug/mL) to 0.012 µg/mL. Only one of the pigs receiving an application of 2 mL had detectable doxorubicinol levels found in the cancellous bone (5 mm and 10 mm) with ranges of 0.002–0.003 µg/mL.

## Discussion

### Main findings

The CarboCell technology was designed to provide a sustained release of doxorubicin from the injected depots to provide continuous delivery of cytotoxic doses directly in cancerous tissues. Hydrophobic ion-pairing was required for doxorubicin to be dissolved in the hydrophobic CarboCell matrix. Importantly, this pairing was demonstrated to enable the sustained release of doxorubicin with maintained anti-cancer activity in murine models.

Having established that CarboCell doxorubicin is released and therapeutically active we proceeded to investigate tissue concentration of doxorubicin in a porcine model, both immediate surrounding and distant from the application site with microdialysis.

The doxorubicin-loaded CarboCell proved easy to inject in the metaphyseal region, however with some oozing from the holes required for injection in the experimental set-up. The possibility to inject in bone using a standard thin needle technology may also allow for injection and dose decoration in soft tissue in cases where spreading or dissemination has occurred or for malignancies other than osteosarcomas [14].

In the porcine model, irrespective of volume (2 or 4 ml), doxorubicin could be measured at least 10 mm from the application site in cancellous bone indicating a good metaphyseal distribution. The systemic spill-over was minimal, and only measurable in three of ten animals.

### Comparison to systemic doxorubicin administration

Until recently, most pharmacokinetic doxorubicin evaluations have been based on plasma or biopsy concentrations. However, plasma concentrations cannot be taken as a surrogate for tissue concentrations, and biopsies cannot distinguish between unbound or bound concentration, intra- or extracellular concentration, and are also limited by poor temporal resolution. Evaluation of release profiles for local devices has often been done in vitro which cannot consider all aspects that can affect the release in vivo [15]. Despite, previous studies reporting adsorption issues, microdialysis has been proven to be a suitable sampling method for doxorubicin, continuously sampling only the unbound active extracellular concentration [13]. This has enabled the evaluation of tissue concentrations after the systemic administration of 150 mg doxorubicin given as both a bolus infusion (15 min) and a continuous infusion (6 h) in a porcine model similar to the one used in the present study [16]. The systemic administration provided very low bone concentrations. On average the mean cancellous C_max_ measured in the present study was a minimum of 75 times higher than the mean cancellous C_max_ after bolus and continuous infusion of 150 mg doxorubicin. As the effect of doxorubicin is believed to be at least partly concentration-dependent, a higher concentration locally (at bone-located tumour site) after local application seems encouraging. Interestingly, it was observed that increasing the CarboCell dosage from 1 to 4 mg/mL neither affected tumour volume nor the probability of survival in the study setup in mice investigating efficacy. The effect of doxorubicin is believed to be exerted both intra- and extracellularly, yet different studies have found varying trigger concentrations and relevant contributions for each mechanism of cytotoxic action [4, 17]. The present results suggest that a saturation level may be reached, where increased concentrations may not demonstrate significant clinical relevance regarding efficacy but are pertinent to side effects. However, a more comprehensive investigation into clinically relevant tumour sizes could provide deeper insights into loco-regional dose distribution and therapeutic efficacy. This information is critically important for future depot injection technologies.

The lower systemic concentration found after local application is also favourable to minimize the risk of systemic side effects. Notably, no systemic doxorubicinol was detected during the study sampling period, which may support the theory of lower risk of cardiotoxicity after local application. One disadvantage of local application is the lack of targeting of metastases (especially micrometastases not visible at diagnosis), circulating tumour cells and potentially dormant cancer cells. The concentration in the investigated bone marrow compartment, which is a common place dormant for tumour cells was low and with poor penetration [18]. Local application may not be seen as a full replacement for systemic administration but as a supplement to increase local efficacy without compromising the risk of systemic toxicity. The local application of doxorubicin can supplement systemic administrations in multi-course therapy, potentiating the local exposure and potentially allowing for a reduced cumulative dose. Encouragingly, despite the well characterized necrotic activity of doxorubicin in cases of extravasation, we did not observe any peri-injection tissue toxcicity, neither after intratumoural injection or subcutaneous injection of CarboCell. This may be attributed to its sustained release profile, ensuring that non-necrotizing doses are delivered at the desired injection sites. The tolerability and therapeutic value of the CarboCell technology has previously been validated for both cancer immunotherapy and antibiotics [8, 19, 20].

### Comparison to other local applications containing doxorubicin

To our knowledge, this is the first study using microdialysis to investigate the local tissue concentrations of doxorubicin following a local in vivo application. However, many different local doxorubicin-containing devices exist. Also, some with additional features such as doxorubicin-loaded bone cement, which can provide some mechanical stability to bone. Cement and PMMA-based materials are however challenged by their solid structure, which potentially hinders sustained release after the material has solidified. This issue is illustrated for antibiotic-eluting cements, where prolonged release of subtherapeutic drug doses is problematic [21–23]. For chemotherapy-loaded cement the total release has been found to be as low as 5-25% (time 0 h-133 days) [22, 24–26]. Use of bone cement has the disadvantage that its therapeutic application needs to be after the surgical removal of tumour tissue (cementoplasty can be given as a palliative treatment [22]), whereas CarboCell can be applied at multiple intratumoral sites (dose decoration) under local anaesthesia before tumour removal surgery, to decrease tumour size, as well as intra- and post-surgery to prevent tumour regression or treat possible residual tumour tissue. Moreover, CarboCell comprises carbohydrate esters, triglycerides and a solvent as excipients that are all biodegradable. In this process, CarboCell is hydrolysed forming disaccharides, glycerol and small molecular fatty acids. Scaffolds are other local application devices which may have advantages such as osteoconductivity and adjustable drug release rate [27, 28]. A potential downside to scaffolds is their structure, which needs to be designed to match the void to provide mechanical support which makes intraoperative placement challenging [29]. On the contrary, CarboCell will conform topographically but do however not possess any mechanical support.

### Limitations

The present study was only partly performed in cancerous tissue, wherefore the potential effect of the tumour microenvironment on the release profile of doxorubicin from the CarboCell (e.g., alternate pH and interstitial fluid pressure [29, 30]) could not be evaluated. Additionally, evaluation of the full release profile needs to be conducted over a longer time as doxorubicin concentrations were still detectable at the end of the 24 h sampling period in pigs.

### Future perspectives

In the present study, the systemic toxicities were considered negligible as plasma concentrations of neither doxorubicin nor doxorubicinol were detectable. However, the local toxicity also needs investigation in future studies. Doxorubicin is used to treat a wide variety of cancers, and the hope is that doxorubicin-loaded CarboCell can reduce the systemic chemotherapeutic burden. Notably, CarboCell represents an innovative liquid technology, characterized by its free-flowing nature, which facilitates surgical injection into virtually any accessible tissue or lesion through standard thin needle injection and aspiration methodologies. This method enables the precise placement of therapeutic depots across targeted lesions, thereby supporting a dose-painting strategy in oncological treatment. CarboCell can be manufactured using simple scalable procedures and cost of goods are low.

## Conclusion

Local application of doxorubicin could be an important supplement to systemic doxorubicin treatment as it has the potential to increase local cytotoxic concentrations and decrease the risk of systemic side effects. Doxorubicin-loaded CarboCell proved easily administrable with maintained antitumoural activity, and it did provide much higher local doxorubicin concentrations when injected in metaphyseal (cancellous) bone compared to those seen after traditional systemic administration. Additionally, only minimal systemic spill-over was observed. This supports further investigation into doxorubicin-loaded CarboCell and the potential of clinical application.

## Electronic supplementary material

Below is the link to the electronic supplementary material.

Supplementary Material 1

## Data Availability

The dataset is available from the corresponding author on reasonable request.
