# Supplementary material for: Injectable sustained local release doxorubicin depot technology– a promising adjuvant to systemic treatment?
Source: Drug Deliv Transl Res. 2025 Apr 3;15(12):4601–10. doi: 10.1007/s13346-025-01841-9 (PMC12619821; doi:10.1007/s13346-025-01841-9)
Supplement: Supplementary file 1 — Supplementary Material 1 [file 13346_2025_1841_MOESM1_ESM.docx]

**Supplementary**


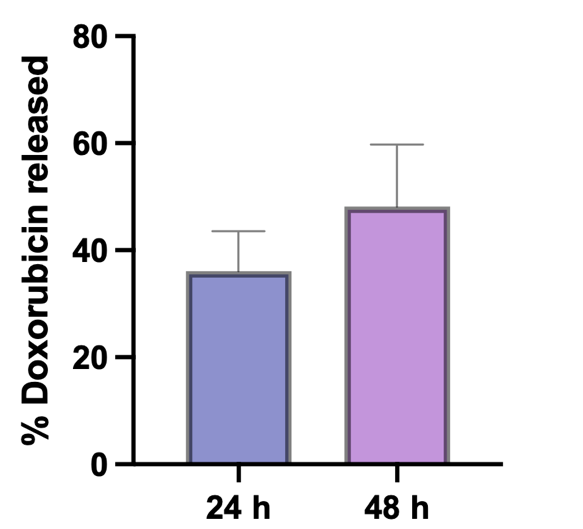


Figure S1: In vivo percentage doxorubicin release of the total depot applied after 24 h and 48 h. A sustained release was seen as 36±13% were released after 24 h and 48±20% after 48 h.
Results are presented as means with standard error of the mean (SEM).

A)

B)


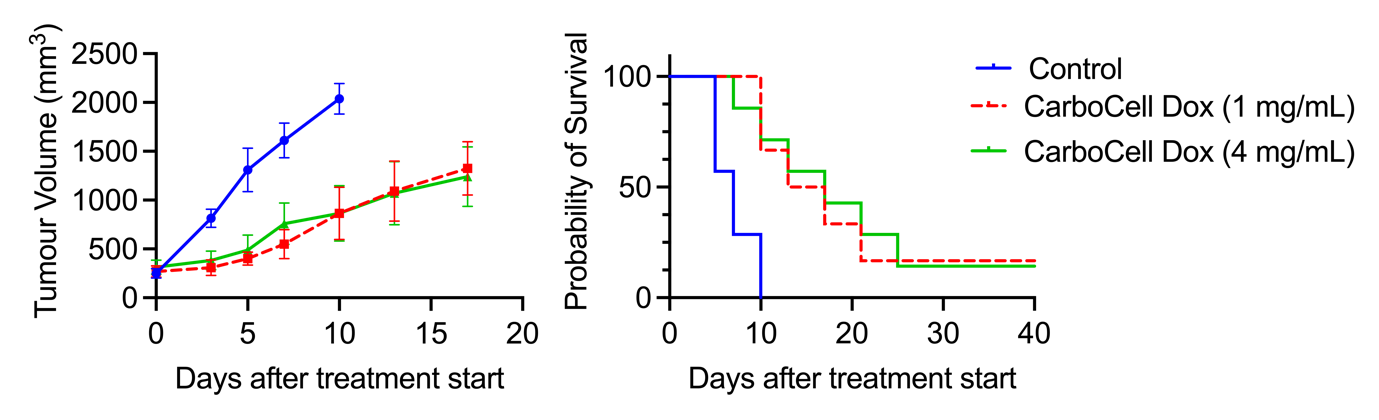


Figure S2: A) Tumour growth curves (volume/mm^3^) and B) probability of survival for mice carrying subcutaneous CT26 tumours and treated with two intratumoral injections of CarboCell doxorubicin (1 mg/mL and 4 mg/mL) at 5 days interval (day 0 and day 5). The therapeutic activity of the CarboCell doxorubicin is evidenced by the reduced mean tumour growth rate in the treatment groups relative to the untreated control. Both CarboCell doxorubicin treatment groups furthermore displayed significantly increased survival time compared to untreated controls (log-rank Mantel Cox, p = 0.0011).

*Analysis of samples from mice*

Approximately 10 μL of pre-injected formulation were dissolved in 1 mL DMSO as reference. Samples were analysed on a Shimadzu Nexera-X with a PDA detector. Samples were injected (5 μL) onto a Waters Terra XBridge BEH C8 column (2.5 μm, 4.6x75mm, temperature 25 °C) at a flow rate of 0.8 mL/min. The solvent system consisted of mobile phase A (5 % MeCN, 0.1 % TFA in water) and mobile phase B (0.1 % TFA in MeCN). The gradient was 0 % B for 1 min, 0 to 100 % B over 5 min, 100 % B for 4 min, 100 % B to 0 % B over 0.5 min, and 0 % B for 1 min. UV detection of Doxorubicin was done at 450 nm and SuBen at 280 nm. The release of doxorubicin was determined using SuBen as internal reference.
